# Tp-e interval, Tp-e/QT, and Tp-e/QTc ratios in patients with primary hyperparathyroidism and their relationship with cardiac arrhythmic events

**DOI:** 10.3906/sag-2107-188

**Published:** 2021-12-09

**Authors:** Yücel YILMAZ, Şaban KELEŞOĞLU, Ferhat GÖKAY, Yasin ŞİMŞEK

**Affiliations:** 1 Department of Cardiology, Kayseri Education and Research Hospital, University of Health Sciences, Kayseri, Turkey; 2 Department of Internal Medicine, Division of Endocrinology and Metabolism, Kayseri Education and Research Hospital, University of Health Sciences, Kayseri, Turkey

**Keywords:** Primary hyperparathyroidism, Tp-e interval, cardiac arrhythmia, electrocardiogram

## Abstract

**Background/aim:**

Primary hyperparathyroidism (PHPT) is an endocrine disorder characterized by hypercalcemia caused by excessive parathyroid hormone (PTH) secretion from the parathyroid gland. PHPT was previously shown to increase cardiac arrhythmias. Besides, new indices, such as the Tpeak-Tend (Tp-e) interval, Tp-e interval/QT interval (Tp-e/QT) ratio, and Tp-e interval/corrected QT interval (Tp-e/QTc) ratio may be associated with ventricular arrhythmias and sudden cardiac death. Therefore, we aimed to investigate the relationship between PHPT and the changes to Tp-e interval, Tp-e/QT ratio, and Tp-e/QTc ratio.

**Materials and methods:**

We carried out the study with 41 patients with PHPT and 40 control subjects. We calculated the Tp-e interval, Tp-e/QT ratio, and Tp-e/QTc ratio of the participants from the V5 derivations on their ECG papers. While we defined Tp-e interval as the distance between the peak and the end of the T wave, Tp-e/QT and Tp-e/QTc ratios were calculated by dividing Tp-e by QT and Tp-e by QTc, respectively.

**Results:**

Total calcium, albumin-corrected calcium, phosphorus, and PTH levels were significantly higher in patients with PHPT. We also found positive correlations between albumin-corrected calcium and PTH levels and Tp-e interval, Tp-e/QT ratio, and Tp-e/QTc ratio (p < 0.001).

**Conclusion:**

Our results suggest that Tp-e may enhance the current knowledge on arrhythmic risk in PHPT patients better than basal ECG. In addition, both high PTH and high calcium levels appear to have the potential to cause arrhythmogenic effects.

## 1. Introduction

Primary hyperparathyroidism (PHPT) is an endocrine disorder characterized by hypercalcemia caused by excessive parathyroid hormone (PTH) secretion from the parathyroid gland and the most common cause of hypercalcemia in outpatient clinics [1–3]. It has been observed more frequently in the last 40 years due to widespread measurements of serum calcium levels [4]. Consequently, PHPT has been becoming a relatively more common endocrine disease with an incidence of 1/1000 [5]. Recently, there has been an increasing interest in cardiac evaluation for patients with PHPT, which may stem from the previous findings that PHPT may increase cardiac mortality and arrhythmias [6,7].

Ventricular recovery and repolarization dispersion on electrocardiogram (ECG) are valuable markers for probable ventricular arrhythmias [8]. The literature hosts several studies suggesting specific ventricular repolarization markers, such as the QT interval and T wave alternans, to be helpful in predicting the arrhythmias [9,10]. Besides, recent studies revealed that novel indices, such as the Tpeak-Tend (Tp-e) interval, Tp-e interval/QT interval (Tp-e/QT) ratio, and Tp-e interval/corrected QT interval (Tp-e/QTc) ratio may be associated with ventricular arrhythmias and sudden cardiac death in various disease groups [11,12].

Ultimately, in this study, we aimed to investigate the relationship between PHPT and the changes to novel arrhythmia markers in PHPT patients: Tp-e interval, Tp-e/QT ratio, and Tp-e/QTc ratio.

## 2. Methods

### 2.1. Sample and research protocol

We recruited and prospectively analyzed 41 randomized patients followed up with a diagnosis of PHPT in Kayseri City Hospital in September 2020. The control group, on the other hand, consisted of 40 randomized, age-gender matching subjects with normal blood PTH levels, who were not suspected of having coronary artery disease based on their medical history, physical examination findings, and ECG and echocardiography results. We recorded the physical examination findings, medical history, and laboratory results of all the participants.

We deemed the exclusion criteria as follows: uninterpretable ECGs (the ones with left bundle branch block, presence of a cardiac pacemaker, and U-waves and negative T-waves in prewarning ECGs), hypertrophic cardiomyopathy, severe valvular disease, coronary artery disease, hypothyroidism and hyperthyroidism, hypokalemia and hyperkalemia, hypomagnesemia and hypermagnesemia, creatinine clearance (CrCl) <60 ml/min, and Body Mass Index (BMI) >30 kg/m^2^. None of our patients were using any medication affecting the QT interval. We obtained written informed consent from all patients, as well as approval from the local ethics committee.

### 2.2. Electrocardiogram analysis

We took all standard 12-lead ECGs in the supine position and at rest using an ECG device (Philips brand) standardized to 1 mV/cm and 25 mm/s paper speed. Then, we scanned and transferred all ECGs into a digital environment. Next, we digitally enlarged ECGs five times for measurement convenience and took the measurements using an electronic caliper (Cardio Calipers software version 3.3; Iconico.com, Philadelphia, PA, USA). Two experienced cardiologists blinded to the clinical data carried out ECG evaluations.

We defined the Tp-e interval as the distance between the peak and the end of the T wave. All Tp-e intervals were measured using the best T wave, if available, and lead V5 in general [13]. Yet, we had to use lead V4 or V6 when lead V5 was not suitable for the analysis.

On the other hand, we adopted the QT interval as the distance from the beginning of the QRS complex to the end of the T wave. It was measured in lead V6, reflecting the transmural axis of the left ventricle the best, and corrected based on the heart rate using Bazett’s formula, that is QTc = QT/√R-R (R-R interval). Finally, we computed Tp-e/QT and Tp-e/QTc ratios by dividing Tp-e by QT and Tp-e by QTc, respectively. The intraobserver and interobserver coefficients of variation were below 5%.

### 2.3. Echocardiography measurements

We performed conventional echocardiographic examinations on the subjects utilizing an M4S-RS (1.5–3.6 MHz) cardiac transducer and a Vingmed System 5 (General Electronic Horten, Norway) echocardiography device. We measured left ventricular diastolic (LVIDd) and systolic (LVIDs) diameters, interventricular septum (IVSWT), and posterior wall (LVPWT) diastolic thicknesses on the parasternal long axis with M-mode echocardiography, following the standards defined by the American Echocardiography Association. Finally, the ejection fraction was calculated using the Teichholz formula [14].

## 3. Statistical analyses

We performed all statistical analyses using the SPSS Statistics software version 21.0 (SPSS Inc, Chicago, IL, USA) for Windows. Initially, we checked the distribution characteristics of the data using the Kolmogorov–Smirnov test. Then, we ran an independent samples t-test for parametric variables. We utilized a chi-square test for univariate analysis of the categorical variables. The variables were reported as means (M) ± standard deviations (SD), whereas the categorical variables were presented as percentages. Finally, we performed relevant correlation analyses (Pearson and Spearman-Brown) to reveal the associations between albumin-corrected calcium and PTH levels and Tp-e interval, Tp-e/QT ratio, and Tp-e/QTc ratio, respectively. In all statistical analyses, we considered a probability value of p <0.05 as significant.

## 4. Results

Table 1 presents the initial clinical and demographic characteristics of the sample. Accordingly, there were no significant differences between the patient and control groups by sex, age, smoking, diabetes, and hypertension (p > 0.05).

The baseline laboratory findings of the subjects are listed in Table 2. Accordingly, total calcium, albumin-corrected calcium, phosphorus, and PTH levels were significantly higher in PHPT patients than healthy controls (p < 0.01 for all). Yet, the groups yielded similar results in other blood parameters. Moreover, the groups did not differ significantly in their echocardiographic parameters (Table 3).

**Figure 1:**
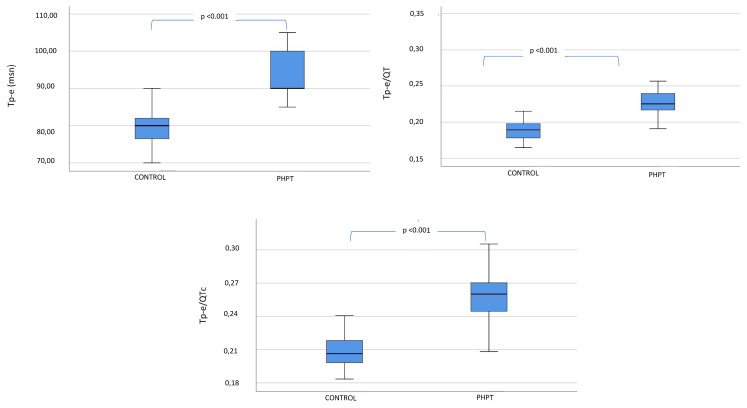
Change of Tp-e interval, Tp-e/QT ratio and Tp-e/QTc ratio between study groups

ECG parameters of the groups are shown in Table 4. Heart rate and QRS duration were similar between the patient and control groups (p = 0.344 and p = 0.220, respectively). Yet, the QT interval was shorter in the patient group than in the control group, contrary to the QTc interval, which was similar between the groups (p = 0.006 and p = 0.810, respectively). Finally, we concluded that Tp-e interval, Tp-e/QT ratio, and Tp-e/QTc ratio were significantly higher in PHPT patients compared to the control group (p < 0.01 in all) (Figure 1).

**Figure 2:**
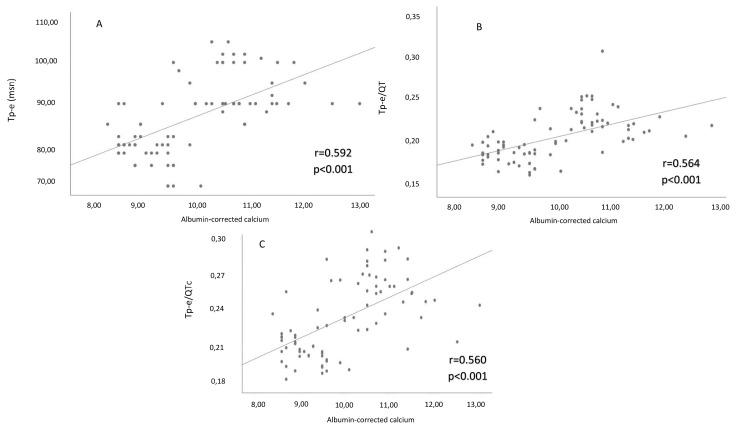
(**A**) Correlation between Tp-e interval and albumin-corrected calcium count. (**B**) Correlation between Tp–e/QTc ratio and albumin-corrected calcium count. (**C**) Correlation between Tp–e/QT ratio and albumin-corrected calcium count

**Figure 3:**
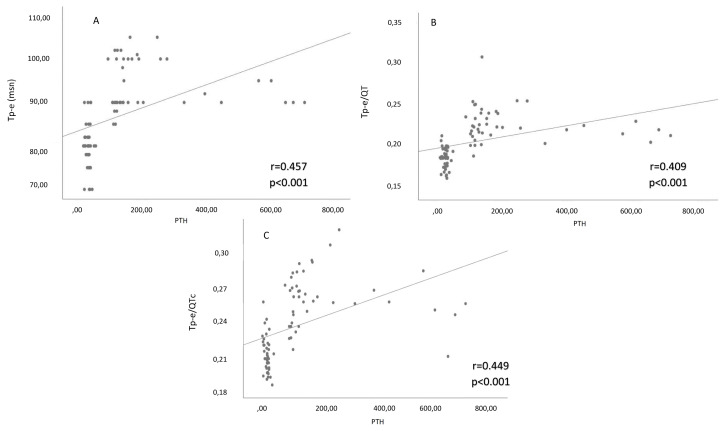
(**A**) Correlation between Tp-e interval and PTH level. (**B**) Correlation between Tp–e/QTc ratio and PTH level. (**C**) Correlation between Tp–e/QT ratio and PTH level

The correlation analysis revealed positive significant associations between albumin-corrected calcium levels and Tp-e interval, Tp-e/QT ratio, and Tp-e/QTc ratio (r = 0592, p <0.001; r = 0.560, p <0.001; r = 0.564, p <0.001, respectively) (Figure 2). There were also positive significant relationships between PTH levels and Tp-e interval, Tp-e/QT ratio, and Tp-e/QTc ratio (r = 457, p <0.001; r = 0.449, p <0.001; r = 0.409, p <0.001, respectively) (Figure 3).

## 5. Discussion

This is the first randomized study in which we demonstrated the prolongation of ECG-detected Tp-e interval to be higher in PHPT patients compared to the healthy subjects.

It is well-known that PHPT is associated with hypertension, cardiac arrhythmias, endothelial dysfunction, glucose metabolism disorder, and metabolic syndrome. Previous research uncovered that both PTH and calcium levels affect cardiomyocytes, the heart conduction system, and smooth vascular and endothelial cells. Excessive secretion of PTH can affect the myocardium and alter repolarization. Although the effects of PTH on the heart were previously thought to be due to hypercalcemia, it is now known that PTH itself causes hypertrophy in cardiac myocytes and vascular smooth muscles independent of calcium levels. In addition, it was reported in the literature that there is a direct link between endothelial dysfunction and PTH [15–19].

Hypercalcemia, including PHPT-induced hypercalcemia, is a risk factor for cardiac arrhythmias [20,21]. Hypercalcemia, which develops in primary hyperparathyroidism, is conventionally accepted to cause shortening of the QT interval, a decrease in the ST segment, and a slight prolongation in the PR and QRS intervals [22]. The shortening of the refractory period due to QT shortening may lead to complex ventricular arrhythmias or sudden deaths [22].

So far, three different cell types have been identified in the ventricle electrophysiologically, namely endocardial, epicardial, and myocardial M cells [23,24]. The peak of the T wave indicates epicardial repolarization, whereas the end of the T wave is shown to overlap with the repolarization of M cells. Hence, the Tp-e interval is the duration of the transmural distribution of repolarization [25]. In the literature, Tp-e was shown to be related to life-threatening arrhythmic events, referring to that Tp-e helps predict the risk of developing arrhythmias [12,26–30]. However, QT and Tp-e intervals widely vary among individuals, and the Tp-e interval is affected by the changes in the heart rate. For this reason, the Tp-e/QT ratio is considered to be more consistent among individuals and their heart rates, regardless of their Tp-e interval values [8].

Pepe et al. and Curione et al. demonstrated that the mean QTc values of the patients with PHPT lie within the normal range but were significantly lower than the mean QTc values of the control group [31,32]. Curione et al. found QT intervals to be shorter in their patients with PHPT; however, the difference was not statistically significant. They also concluded QT intervals to be within the normal range [33]. In our study, we also found that QT intervals were shorter in the patient group than in the control group. In addition, similar to the findings of the studies above, we could not find any significant differences between QTc intervals of the patient and the control groups; they were within normal limits, as well. Such a finding may be due to the mild hypercalcemia in our patients since PHPT patients with higher calcemic levels are known to have shorter QT intervals.

Our findings showed that Tp-e interval, Tp-e/QT ratio, and Tp-e/QTc ratio were higher in patients compared to the control group. Yamaguchi et al. suggested that the Tp-e/QT ratio is a better predictor of torsade de pointes than the QTc interval [25]. Watanabe et al. demonstrated that longer Tp-e intervals are associated with spontaneous ventricular tachycardia. Besides, Shimizu et al. reported that Tp-e/QT ratios were higher in patients developing sudden cardiac death than those who did not [29, 34]. Hevia et al. revealed that the incidence of recurrent cardiac events is significantly higher in patients with increased Tp-e ranges [12]. Ventricular arrhythmias in PHPTwere generally reported in the form of case reports in the literature; however, we could not encounter follow-up studies of the long-term cardiovascular consequences of PHPT. Nilsson et al. and Pepe et al. reported an increase in ventricular extrasystolic beats (VPB) in patients with PHPT [31,35]. They demonstrated the predictor of VPBs in PHPT patients to be serum total calcium levels [31]. In our follow-up study, we concluded our patients to have increased Tp-e interval, Tp-e / QT ratio, and Tp-e / QTc ratio, which are considered strong predictors of cardiac arrhythmia. Along with the results reported in the literature, our results suggest that patients with PHPT are at risk for severe ventricular arrhythmia and sudden cardiac death.

On the other hand, we found correlations between Tp-e interval, Tp-e/QT ratio, Tp-e/QTc ratio, and total calcium levels and PTH levels in the patients. Curione et al. demonstrated that the QT interval remained unchanged even though serum calcium levels improved following parathyroidectomy [32]. The correlation between PTH and Tp-e interval, Tp-e/QT ratio, and Tp-e/QTc ratio in our study suggest that arrhythmogenesis mechanisms may be associated with both PTH and calcium levels. PTH has chronotropic effects on animal models and affects coronary blood flow and contraction [36]. PTH may cause both hypertrophy and necrosis by directly affecting the cardiac myocytes, and hypercalcemia may also affect Tp-e and QT durations by electrically shortening the plateau phase of the cardiac action potential and the effective refractory period [37–39]. In addition, PTH has been shown to increase systemic inflammation markers [40]. Inflammation markers may also have affected Tp-e and QT by affecting cardiac repolarization. Our results indicate that arrhythmogenesis mechanisms may be associated with both PTH and calcium levels.

To date, the research with PHPT patients has investigated the risk of cardiac arrhythmia through the QT interval. It is not veiled that shortening the duration of the QT interval is associated with an increased risk of arrhythmia and sudden cardiac death. It is a matter of debate whether the risk of arrhythmia increases in PHPT patients with a regular QT interval. In this study, we demonstrated that ventricular repolarization showed an abnormal distribution with an increase in Tp-e interval, Tp-e/QT ratio, and Tp-e/QTc ratio in PHPT patients with normal QT interval regardless of the QT interval and that this group of patients is more susceptible to future ventricular arrhythmias and sudden cardiac death.

The present research bears few limitations. First, we had no information on how long the patients had lived with PHPT and hypercalcemia before the diagnosis. Second, we did not know the threshold values of PTH or calcium levels, which may lead to significant changes in the heart, as well as the duration for how long PTH or calcium levels must remain high for these significant changes to occur. Most of the studies to that effect included patients with symptomatic diseases. Therefore, an intervention in a presymptomatic stage and a lower level of hypercalcemia may perhaps alter the disease trajectory. Finally, although values such as Tp-e interval, Tp-e/QT ratio, and Tp-e/QTc ratio are secondary markers of arrhythmia, we did not perform long-term clinical follow-ups and rhythm Holter follow-ups to detect the development of arrhythmia.

## 6. Conclusion

Our results suggest that Tp-e may provide more information about arrhythmic risk in PHPT patients than basal ECG. In addition, both higher PTH and calcium levels appear to have the potential to cause arrhythmogenic effects. A larger sample and long-term follow-up studies are needed to observe the clinical consequences of these findings and, perhaps, to come to a decision of an earlier medical treatment or surgery.

## Figures and Tables

**Table 1:** Baseline clinical findings and demographic characteristics of the sample.

Variables	Control group (n = 40)	PHPT (n = 41)	p value
Age (years)	57.52 ± 11,10	57.53 ± 12,35	.996^1^
Male/female	37/3	37/4	.718^1^
HT	19 (47.5%)	19 (46.3)	.917^2^
DM	7 (17.5 %)	7 (17.1%)	.959^2^
Smoke	1 (2%)	1 (2%)	.986^2^
SBP (mm/hg)	119.50 ± 10.20	122.60 ± 11.50	.201^1^
DBP (mm/hg)	73.70 ± 7.20	74.70 ± 6.30	.507^1^
BMI	57.52 ± 11.10	57.53 ± 12.35	.680^1^

PHPT; Primary hyperparathyroidism, DM: Diabetes Mellitus, HT: Hypertension, SBP: Sistolic Blood Pressure, DBP: Diastolıc Blood Pressure, BMI; Body Mass Index, Data are expressed as mean ± standard deviation for normally distributed data and percentage (%) for categorical variables.

P^1^: Independent samples t-test.

P^2^: Chi-square.

Statistically significant (p < 0.05).

**Table 2:** Comparison of baseline laboratory measurements of the sample.

Variables	Control Group (N = 40)	PHPT (N = 41)	p value
Glucose (mg/dL)	94.6 ± 10.90	97.8 ± 13.50	.237
Creatinin (mg/dL)	0.84 ± 0.19	0.83 ± 0.15	.730
AST (U/L)	19.8 ± 6.30	19.6 ± 6.80	.920
ALT (U/L)	19.05 ± 6.80	20.95 ± 10.4	.355
Albumin	3.96 ± 0,50	4.53 ± 0.30	.001
Total calcium (mg/dL)	9.53 ± 0.36	11,29 ± 0.73	.001
Albumin-corrected calcium (mg/dL)	9.13 ± 0.44	10.87 ± 0.69	.001
Phosphorus (mg/dL)	3.72 ± 0.41	2.57 ± 0.50	.001
PTH	35.20 ± 8.16	226.91 ± 172.51	.001
D Vitamin	20.11 ± 7.7	19.66 ± 7.98	.800
Platelet (x10^3^/mm^3^)	264.2 ± 68.0	259.2 ± 62.60	.729

PHPT: Primary hyperparathyroidism, PTH: Parathyroid Hormone. Data are expressed as mean ± standard deviation for normally distributed data and percentage (%) for categorical variables.

p value: independent samples t-test.

* Statistically significant (p < 0.05).

**Table 3:** Echocardiographic characteristics of the sample.

Variables	Control Group (N = 40)	PHPT (N = 41)	p value
LVEDD	4.68 ± 0.41	4.80 ± 0.44	.198
LVESD	3.11 ± 0.41	3.05 ± 0.32	.463
IVSD	1.05 ± 0.97	1.05 ± 0.18	.871
PWD	1.01 ± 0.84	1.01 ± 0.17	.915
LVEF	62.3 ± 3.1	63.3 ± 4.1	.214

PHPT; Primary hyperparathyroidism, LVEDD: Left Ventricular End Diastole Diameter, LVESD: Left Ventricular End Systole Diameter, IVSD: İnterventricular Septal Diameter, PWD: Posterior Wall Diameter, LVEF; Left Ventricular Ejection Fraction, Data are presented as mean ± standard deviation for normally distributed data and percentage (%) for categorical variables.

p value: independent samples t-test.

Statistically significant (p < 0.05).

**Table 4:** Electrocardiographic characteristics of the sample.

Variables	Control Group (N = 40)	PHPT (N = 41)	p value
Heart rate (beat/min)	75.9 ± 8.5	78.1 ± 12.07	.344
QRS duration (ms)	85.0 ± 8.5	82.6 ± 8.6	.220
QT interval (ms)	380.3 ± 20.7	365.2 ± 27.4	.006*
QTc interval (ms)	421.2 ± 15.4	412.8 ± 25.9	.081
Tp-e (ms)	79.5 ± 5.2	94.09 ± 5.7	.001*
TPe/QTc ratio (ms)	0.18 ± 0.01	0.22 ± 0.02	.001*
TPe/QT ratio (ms)	0.20 ± 0.15	0.25 ± 0.23	.001*

PHPT; Primary hyperparathyroidism. Data are shown as mean ± standard deviation for normally distributed data and percentage (%) for categorical variables. Tp-e= T wave interval from peak to end, c = corrected, P value: independent samples t-test.

* Statistically significant (p < 0.05).

## Data Availability

Data and any supplementary material related to this article can be obtained from the corresponding author on request.
